# Classification of Protein-Binding Sites Using a Spherical Convolutional Neural Network

**DOI:** 10.1021/acs.jcim.2c00832

**Published:** 2022-11-07

**Authors:** Oliver B. Scott, Jing Gu, A.W. Edith Chan

**Affiliations:** Division of Medicine, University College London, Gower Street, LondonWC1E 6BT, U.K.

## Abstract

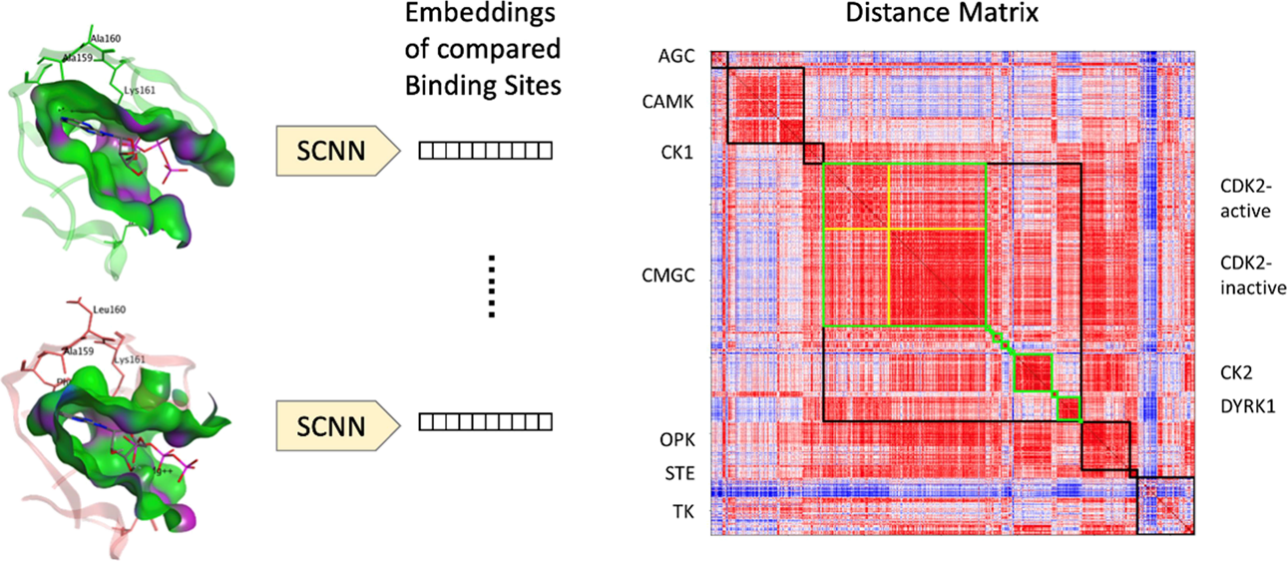

The analysis and comparison of protein-binding sites aid various applications in the drug discovery process, e.g., hit finding, drug repurposing, and polypharmacology. Classification of binding sites has been a hot topic for the past 30 years, and many different methods have been published. The rapid development of machine learning computational algorithms, coupled with the large volume of publicly available protein–ligand 3D structures, makes it possible to apply deep learning techniques in binding site comparison. Our method uses a cutting-edge spherical convolutional neural network based on the DeepSphere architecture to learn global representations of protein-binding sites. The model was trained on TOUGH-C1 and TOUGH-M1 data and validated with the ProSPECCTs datasets. Our results show that our model can (1) perform well in protein-binding site similarity and classification tasks and (2) learn and separate the physicochemical properties of binding sites. Lastly, we tested the model on a set of kinases, where the results show that it is able to cluster the different kinase subfamilies effectively. This example demonstrates the method’s promise for lead hopping within or outside a protein target, directly based on binding site information.

## Introduction

The analysis of the three-dimensional (3D) structures and characteristics of proteins, especially their binding sites, is vital for the understanding of their biological function, as well as for drug development. Computer technology is widely used in the drug discovery process, e.g., small molecular virtual screening,^1^ structure-based drug design, and docking of small molecules and proteins. Analysis of ligand–protein complexes in the Protein Data Bank (PDB)^2^ has shown that most ligands interact with specific binding sites on the targeted proteins; hence, each binding site has a set of unique characteristics/properties or biological functions that distinguish it from other cavities on the protein surface.^3^ These properties enable the binding of specific ligands from the thousands of biomolecules that are found in the complex biological environment of a living cell.

The characteristics of binding sites can be divided into two main categories: shape-related properties (e.g., volume, depth, geometric features, and flexibility) and physicochemical properties (e.g., electrostatic potential, hydrophobicity, hydrogen bond potential, and aromaticity).^4^ Analysis of ligand-binding sites has significant applications in fields of molecular docking, drug–target interactions, compound design, ligand affinity prediction, and molecular dynamics.^5^

The significant conservation of the geometric and physicochemical properties of binding sites has enabled the development of binding site identification algorithms using protein structure without the requirement for ligand structural information.^6−10^ Comparison of the dissimilarities between evolutionarily related binding sites has been applied to study how small molecules target specific proteins. Conversely, similarities in the binding sites of unrelated proteins have also been identified.^11,12^ Such local binding site similarities can be helpful in the prediction of drug promiscuity,^13−15^ drug repurposing,^16,17^ protein function classification, and the determination of off-target side effects.^18^ It can also help at the start of a drug discovery campaign, suggesting potential compound classes or molecular scaffolds from matched protein targets, especially if they are not evolutionarily related.

The main hurdle when evaluating binding site similarity is the formulation of a quantitative definition of similarity. Unfortunately, no unique definition exists,^18^ predominantly due to intrinsic subjectivity. Predictably, the lack of a concrete definition has led to the development of a large selection of algorithms all varying with respect to the representation, featurization, and numerical evaluation of similarity.^14^ These methods tend to be optimized based on small, hand-crafted datasets introducing various biases into the calculations.

Recently, protein structural modeling has been greatly influenced by deep learning techniques due to its superior pattern recognition abilities,^19^ with applications ranging from protein structure prediction,^20^ protein–protein interaction prediction,^21,22^ protein–ligand-binding affinity prediction,^23−25^ and binding site identification.^10,26,27^ Deep-learning-based methods give a good fit for protein modeling as biases associated with traditional analytical approaches are removed.^28,29^

Convolutional neural networks (CNNs) have proved successful in image processing mainly due to their equivariance to translations in Euclidean space.^30^ The most natural adaptation of deep learning to 3D is thus to extend 2D-CNNs using collections of 3D filters and voxel-grid representations of 3D objects. This type of volumetric model has been applied to protein-structure modeling tasks and more specifically to binding-site-related objectives. Pu et al. developed DeepDrug3D,^31^ a 3D-CNN-based model, with a demonstrated high accuracy to classify binding sites based on the type of ligand they interact with (nucleotide and heme). While features from intermediate layers may potentially be used for similarity analysis, differentiation of unseen protein structures is not meaningful due to the directed nature of the learning objective. Simonovsky and Meyers introduced DeeplyTough,^29^ a 3D-CNN-based model for pocket comparison. The learning objective is framed as a metric-learning task, taking inspiration from computer-vision techniques, where (binary) ground truth relationships are defined based on shared interactions with a structurally similar ligand. The authors used the daTaset tO evalUate alGoritHms for binding site Matching (TOUGH-M1) dataset^32^ for training since it represents the largest binding-site pair dataset constructed to date. This method performs consistently well across the Protein Site Pairs for the Evaluation of Cavity Comparison Tools (ProSPECCTs) benchmark datasets.^11^

In general, 3D-CNNs have limitations inherent in their design, especially in terms of computational efficiency, where cost increases to the third power. Since voxels represent both occupied and unoccupied regions of the binding site, convolutions are performed over large areas of empty space. The large parameter space of 3D-CNNs also makes them susceptible to overfitting.^33^ As a result, most protein-modeling tasks use relatively shallow networks compared to state-of-the-art image processing networks. Furthermore, despite translational equivariance, invariance to other deformations, such as rigid rotations, is often addressed through costly data augmentation. Despite these inherent limitations, little emphasis has been placed on the exploration of alternative representations and models for binding-site-based learning objectives. The MaSIF model^22^ used protein surface representations, applying geodesic convolutions to overlapping patches with a fixed geodesic radius. The process involves sampling a fixed number of surface patches and mapping these to a geodesic polar coordinate system using a multidimensional scaling algorithm. The complexity of the model, however, limits its potential use in large-scale applications.

Recently, a new paradigm of neural network architecture has been developed, leveraging spherical representations of data such as panoramic images, brain activity data, and LIDAR scans. Numerous spherical CNNs have been developed^34−38^ to infer labels or variables from these representations, with the advantage of equivariance to the rotation group SO(3). The approach has shown success in the field of computer vision, demonstrating highly competitive results for shape classification and retrieval, especially when considering arbitrary rotations where other models fail to generalize. It is worth noting that the spherical representation of 3D objects was used in shape analysis even before the advent of deep learning,^39,40^ due to the sphere’s inherent invariant properties. Indeed, spherical representations have been leveraged for local protein environment similarity evaluation, binding site classification and retrieval, and protein–ligand interaction prediction.^41−45^ These approaches commonly use spherical harmonic decomposition of spherical functions, representing geometric and physicochemical properties.

In our study, we use a graph-based spherical CNN proposed by Perraudin et al.^38^ for binding-site-related tasks. The tasks include a classification and metric learning objective, trained and evaluated using established datasets: daTaset tO evalUate alGoritHms for binding site Classification (TOUGH-C1)^31^ and TOUGH-M1,^32^ respectively. In the metric learning case, the model is used to compute rotationally invariant binding site descriptors which can be evaluated efficiently in a pairwise manner using the Euclidean distance metric. We further evaluate the trained model on the ProSPECCTs benchmark dataset for analysis of generalizability to unseen data and for convenient comparison with existing algorithms. Finally, we carry out a large-scale structure comparison of protein kinase ATP-binding sites using our trained model. The results show that our model can use local features to reveal similarities within different kinase families. We observe similarity trends within subfamilies, corresponding to active and other states,^46^ emphasizing the sensitivity of our models to the biological features of the protein structures. The results demonstrate the potential of alternative binding site representations and deep-learning models. We hope that our work inspires the exploration of further representations and their use in protein-structure applications.

## Datasets

Our model, which we will refer to as BindSiteS-CNN, computes vector representations of protein-binding sites from shape and physicochemical features mapped to spherical projections of binding site surfaces using a spherical CNN. The model is trained with two different objectives: binding site classification and binding site representation learning. The classification task is trained and validated using the TOUGH-C1 dataset which contains protein-binding sites labeled by the type of ligand they bind. The representation learning objective is trained with the binding site pair dataset TOUGH-M1 and validated using the ProSPECCTs binding site similarity benchmark datasets. Finally, a case study is performed using a set of protein kinases.

Steroids from the TOUGH-C1 were not included as controls during the training process. There were 7117 unique UniProt codes in TOUGH-M1 and 67 in the kinase set, with 30 overlaps between the two sets. As we were comparing against previously published results using the ProSPECCTs dataset, which is a common benchmark, we did not remove the overlaps to allow for this comparison. Table 1 summaries the descriptions of the datasets used.

**Table 1:** Descriptions of Datasets

dataset	ligand type for the subset	number of structures	use	classification objective	representation learning objective	ref
TOUGH-C1	nucleotide	1553	training	used	not used	(32)
	heme	596	“	“	“	
	control	1946	“	“	“	
	steroid	69	validation	used	x	(32)
TOUGH-M1	selected drug-like molecules	7524	training	x	used	(33)
ProSPECCTs	varies according to the subset	varies according to the subset	validation	x	used	(12)
kinases	inhibitors such as ATP and small molecules	1264	case study	x	used	Table S1

### Classification Objective: TOUGH-C1

**Figure 1:**
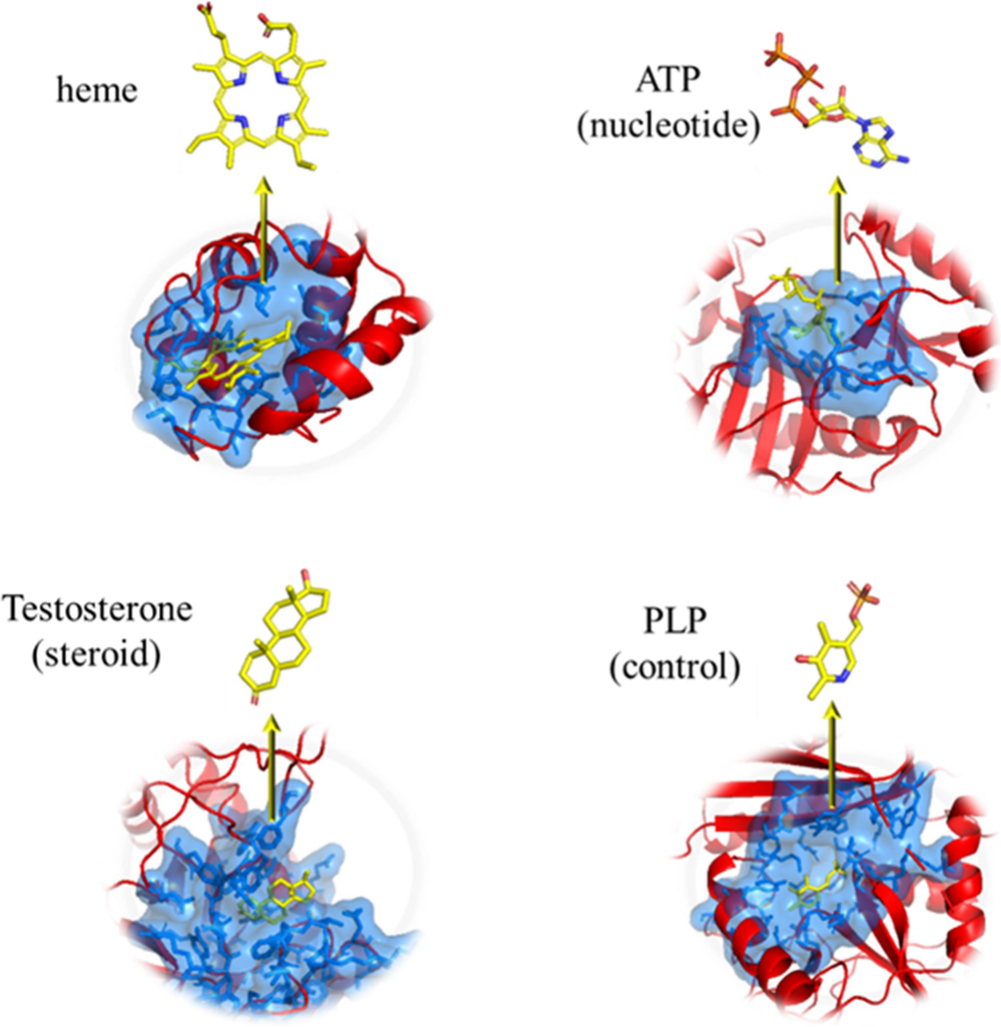
Four classified subsets of TOUGH-C1. The ligands (yellow) and binding sites, as well as binding pockets (blue) of the example proteins (red), are shown. PDB IDs: heme-1A2S, nucleotide-1A0I, steroid-1AFS, and control-1A3G. The control group represents an ensemble with ligands that are different from the three other subsets.

TOUGH-C1 is a dataset for training and cross-validating protein-binding site classification models. It consists of binding sites labeled with the type of ligand they interact with: either nucleotide, heme, or control. A further validation set consisting of steroid-binding sites forms an external validation set. Binding sites labeled “control” form a subset of the TOUGH-M1 dataset containing only proteins with a sequence identity ≤40% and a template modeling (TM)-score^47^ ≤0.5 to any nucleotide-, heme-, and steroid-binding protein. The TM score is an evaluation of the global structure similarity between a pair of proteins. The value ranges from 0 (totally dissimilar) to 1 (identical). Control proteins are further filtered if they contain ligands with a Tanimoto coefficient > 0.5 to any ligand in the other subsets. The resultant dataset contains 1553 nucleotide-binding, 596 heme-binding, and 69 steroid-binding complexes, plus a control set with 1946 complexes (Figure 1).

### Representation Learning Objective

#### TOUGH-M1

TOUGH-M1 is a large dataset containing over one million labeled binding site pairs. The dataset represents the largest, most balanced binding site similarity benchmark to date—two ideal properties for training machine-learning-based algorithms. During construction, important factors such as noncovalent binding of the protein to the ligand, drug-like properties of the ligand, sequence identities of the proteins, and similarities of the ligands were also considered. The positive subset of TOUGH-M1 contains 505,116 protein pairs that are structurally dissimilar but with chemically similar ligands. The negative subset contains 556,810 protein pairs, where both protein structure and bound ligands are dissimilar.

#### ProSPECCTs

ProSPECCTs is an extensive collection of datasets built for the performance evaluation of binding site similarity comparison tools. It contains 10 benchmark sets, each crafted to test a different aspect of binding site similarity evaluation and to identify strengths and weaknesses within a given algorithm. As the scoring is unlikely to be consistent across all the datasets, the tools should be selected according to the given application and the information available in the various benchmarks.

#### Protein Kinases

Protein kinases are among the most studied druggable targets, particularly in the field of oncology for the discovery of anticancer therapeutics.^48^ Their active site, the ATP-binding site, exhibits remarkable structural variation across the proteins of this family, despite them all binding the same substrate. The medicinal effort during the past 20 years has seen a high diversity of synthetic ATP mimetics/inhibitors for different kinases. A drug’s specificity and selectivity are very important when designing and optimizing drugs toward specific targets during the drug discovery process.^49^ Especially challenging is the active form of the pocket. Many successful efforts have been published attempting to classify the structures and their interactions with different inhibitors.^48,50^

Here, we compared the active conformations of the ATP-binding sites using a pretrained BindSiteS-CNN to test if our method can classify kinases using learned representations. The Molecular Operating Environment (MOE) software contains a well-defined protein kinase database, classified according to a widely accepted definition.^50^ For our study, binding sites containing complete activity annotation were selected for comparison. The entries from the MOE kinase set were selected if they contained a “DFG” motif (responsible for kinase activation) or an “alpha C” motif (defined by the spatial position of Lys72 which is secured by a salt-bridge from Glu91 and which can be “in” or “out”). In addition, only PDB structures of the whole protein, containing ligands and having active state information, were retained (1264 structures in total). The proteins were labeled based on the group, family, and subfamily for further analysis. This dataset is provided in the Supporting Information (Table S1). Using cyclin-dependent kinase 2 (CDK2) as an example: the definition of labeling follows:

Group: CMGC contains cyclin-dependent kinase (CDK), mitogen-activated protein kinase, glycogen synthase kinase (GSK3), and CDC-like kinase.
Family: CDK is a member of the cyclin-dependent kinase family (CDK).
Subfamily: CDK2.

## Methods

### Binding Pocket Surface Preparation

#### Pocket Generation

Ligands tend to interact with proteins in depressed regions of the molecular surface, referred to as pockets or clefts. In enzymes, the largest pocket region commonly contains the active site.^51^ Many algorithms have been developed to identify these regions,^6,7,9,52,53^ using purely surface geometry or in combination with various chemical properties. Here, SURFNET^6^ was used to locate pockets through the placement of spheres between pairs of protein atoms such that the radius of the sphere does not penetrate the van der Waals radius of any other atom. Clusters of overlapping spheres represent the 3D shape of each pocket. The surface of these spheres delineates a negative imprint, or image, of the pocket.

#### Pocket Filtering

One problem with the SURFNET algorithm is that it often overestimates the size of ligand-binding regions^52^ and hence is not useful for shape comparisons. Morris et al.^42^ mitigated this issue by filtering the SURFNET spheres based on the conservation value of the nearest residue, where conservation is calculated using the ConSurf algorithm.^54^ However, this process is computationally expensive and requires being able to obtain suitable multiple sequence alignments. Instead, we filter the spheres using protein atoms that define the ligand-binding region of the pocket based on three criteria. First, if a ligand is co-crystallized, protein atoms are selected within a radial threshold **r** of the ligand’s heavy atoms; second, if multiple ligands are co-crystallized in different PDB entries, the atom selection involves taking an ensemble of all the protein atoms in the different structures within a radial threshold **r** of the ligands’ heavy atoms; third, if a protein has no ligand co-crystallized, binding-site atoms are calculated using FPocket.^9^ A convex-hull (the smallest envelope containing all points) is built from the 3D coordinates of the selected protein atoms, and all spheres outside of its volume are discarded. The value of the radial threshold **r** was selected by a process of trial and error. A value of 6 Å gave a reasonable representation of the pockets.

#### Pocket Surface Calculation

With the filtered SURFNET pocket spheres, the next step is to calculate the triangulated surface of those spheres, upon which a set of features can be projected. MSMS^55^ is used to generate the solvent excluded surface of the spheres with a probe radius of 1.5 Å and a triangulation density of three vertices per Å^2^. A basic clean-up removes degenerate faces, duplicate faces, infinite values, and unreferenced vertices. Four iterations of Laplacian smoothing^56^ are also applied to remove noise from the surface generation process.

### Property Calculation

The vertices of the computed pocket surface mesh are enriched with physicochemical information describing the hydrophobicity, electrostatic potential, and interaction-based classification of surface-exposed atoms lining the pocket.

#### Interaction-Based Classification: Pseudocenters

**Figure 2:**
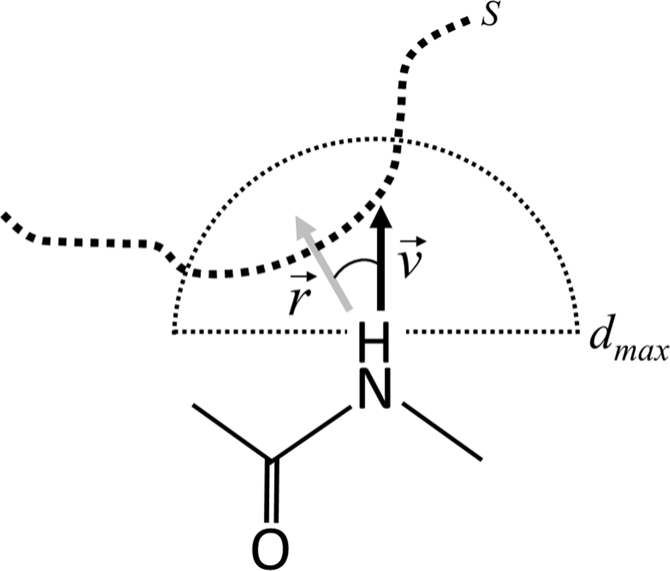
Exposure of an individual physicochemical property is determined using the vectors *v* and *r*. The vector *v* represents the direction of exposure where, in the case of the backbone N-terminal nitrogen (donor), the vector is constructed along the projected N–H is by bisecting the angle formed by the peptide bond (C–N–Cα). The vector *r* is a normalized summation vector calculated from all vectors pointing from the nitrogen atom to all neighboring surface (S) vertices within a spherical region defined by the radius *d*_max_. The angle between the two vectors, ∠ (*v*, *r*), determines whether the pseudocenter projects its property into the binding site cavity with the potential to form an interaction with a putative ligand (Table 2). Adapted from ref (4).

For each pocket lining residue, a set of generic pseudocenters is defined to represent five properties essential for forming interactions: hydrogen-bond donor (DON), acceptor (ACC), mixed donor/acceptor (DAC, e.g., side-chain nitrogen atoms in histidine ND1/NE2), and aliphatic (ALI) and aromatic/PI (ARO, PI is any kind of pi interaction). The pseudocenters are constructed on binding site residues centered at locations defining particular physicochemical features.^4,57^ Two vectors, *v* and *r*, are assigned to each center, where *v* represents the average vector along which an interaction could be formed, and *r* is a normalized summation vector, aggregated from all vectors pointing from a particular pseudocenter to all surface points of a sphere of radius 3 Å. The computed angle between *v* and *r* is used as a criterion for filtering. Table 2 summarizes the cut-offs used for four of the five pseudocenter classifications, and Figure 2 illustrates the procedure. Aliphatic centers (ALI) are not considered in the filtering procedure as interactions are assumed to be isotropic through van der Waals forces. Once constructed and filtered, pseudocenters are projected onto the pocket surface mesh vertices, where the closest pseudocenter to each vertex is considered. If there is no corresponding pseudocenter within a 3 Å radius, the vertex is marked as NULL assuming that the vertex occupies the opening of the pocket. The final assignments are one-hot encoded into a numerical vector representing the pseudocenter classification. The DAC pseudocenter is encoded as both DON and ACC rather than having its own class, resulting in a vector of four binary values.

**Table 2:** Cut-Offs Used for Four of the Five Pseudocenter Classifications

pseudocenter	type	cutoff (°) for the angle between vectors *r* and *v*
donor	DON	100
acceptor	ACC	100
donor/acceptor	DAC	120
aromatic/PI	ARO	100

#### Hydrophobicity

Hydrophobicity is commonly quantified using various residue or atomic level scales, where higher/lower values correspond to increased or decreased hydrophobicity. The values for an atomic level hydrophobicity scale^58^ and the partial charges for nonpolar atoms and polar atoms are −1 (partial charge 0–0.25) and +1 (partial charge >0.25), respectively. Surface vertices were assigned a hydrophobicity value based on the average hydrophobicity of all atoms within a sphere of radius 4.5 Å, where the hydrophobicity values are scaled by their distance to the corresponding vertex.

#### Electrostatic Potential

Proteins were protonated using the REDUCE software.^59^ Electrostatic and partial charges were calculated with PDB2PQR.^60^ APBS (v.3.0)^61^ was used to calculate Poisson–Boltzmann electrostatics for each protein, using default parameters. Charge values were interpolated at each vertex using Multivalue, provided within the APBS software suite. Charges were capped to ±30 and normalized between −1 and 1.

### Architecture: Spherical CNN

Convolutions on the sphere are not as straightforward as convolutions in the Euclidean domain due to nonuniform samplings of the sphere. Spherical CNNs thus commonly implement convolutions on the sphere by realizing them in the spherical harmonic domain.^30,35^ While these operations are equivariant to rotations, they are computationally expensive. A different approach models the sampled sphere as an undirected graph connecting pixels according to the distance between them, where the distance between any two pixels approximates the geodesic distance between them.^36,38,62^ Laplacian-based graph convolutions applied to spherical graphs approximate spherical convolutions with the benefit of increased efficiency but at the cost of exact equivariance. The DeepSphere architecture^62^ uses the graph CNN proposed by Defferrard et al.,^63^ giving competitive performance and a reduced cost for 3D object recognition. SHREC’17 shape retrieval contest data^64^ and the DeepSphere architecture were used for this method.

#### Sampling

For construction of a discretized sphere, a sampling scheme  must be used containing *n* points assigned with the values of the signals to be processed. Due to the absence of a uniform sampling on the sphere, many sampling schemes have been proposed, each with different trade-offs. They include the equiangular,^65^ Hierarchical Equal Area isoLatitude Pixelization (HEALPix),^66^ and icosahedral samplings. The HEALPix sampling scheme, used in this work, is based on the hierarchical subdivision of a rhombic dodecahedron, producing a discretization of the sphere where each pixel covers equal area. The lowest possible resolution corresponds to the base surface partition, with twelve equal-area pixels (*N*_pix_). The resolution of the sampling changes according to the function: *N*_pix_ = 12*N*_side_^2^ such that at *N*_side_ = 16, *N*_pix_ = 3,072.

#### Graph Construction

From the HEALPix sampling, a weighted undirected graph is constructed , where  is the set of vertices ,  is the set of edges, and *w* is the weighted adjacency matrix. In the corresponding graph, pixels are represented as vertices  and each vertex *v_i_* is connected to its neighboring *k* vertices *v_j_*, forming a set of edges . The weighted adjacency matrix  is then constructed as follows:
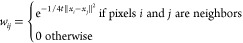
where *x_i_* is a vector encoding the 3D coordinates of pixel *i*, and *t* is a kernel width optimized to minimize equivariance error given *k* neighbors and the sampling resolution *N*_pix_. A weighting scheme is important for equivariance since the distances between pixels in each sampling will not be equal. For full details of the weighting scheme, the reader is directed to Defferrard et al.^62^

#### Graph Convolutions

The graph convolution introduced by Defferrard et al. on spherical signals is defined as follows:
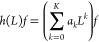
where *K* is the polynomial order corresponding to the filter size, *a_k_* is the coefficient optimized during training, and *L* is the graph Laplacian matrix . The combinatorial Laplacian is defined as *L* = *D* – *W*, where *W* is the weighted adjacency matrix *W* = (*w_ij_*) and *D* is the diagonal degree matrix *D* = (*d_ii_*) and *d_i_* = ∑*_j_W_ij_* is the weighted degree of *v_i_*. *L^k^* captures *k*-neighborhoods, where the entry (*L^k^*)_*ij*_ indicates the sum of length *k* weighted paths between vertices *v_i_* and *v_j_*, where the weight of a path is the multiplication of the edge weights along that path. Filtering with a polynomial convolution kernel can thus be seen from the vertex domain as a weighted linear combination of neighboring vertices. The overall cost of the convolution reduces to  through recursive application of *L*, compared to  for the SHT-based approaches.^30,35^

#### Pooling

Since the HEALPix sampling scheme is intrinsically hierarchical, down-sampling pixels is naturally simple since each subdivision divides a cell in an equal number of subcells. To down-sample the graph, aggregation of subcells with a permutation invariant function, such as the maximum or the average, is used to summarize information, producing a coarser graph.

### Spherical Feature Maps

The classification and retrieval of 3D shapes is a task that requires invariance to rotations. Proteins and their associated binding sites have no canonical orientation: rigid (isometric) transformations do not change their nature. Protein surfaces are commonly represented as triangulated meshes or point clouds, which are difficult to process in a rotation-invariant manner. We propose to project the pocket imprint surface onto a property attributed spherical map, which naturally allows rotation invariant treatment.

The calculation of spherical maps begins with scaling the pocket surface to fit inside the unit sphere, and a ray-casting technique is utilized to project the pocket to the sphere. Rays emanate from pixels sampled on the sphere’s surface toward the origin, and the point of intersection is recorded. From the point of intersection, a depth map is created using the distance from the surface, and the cos and sin of the angle formed between the ray and the surface-normal (face) forms two normal maps, describing the shape of the pocket surface.

Physicochemical maps can then be calculated by aggregating properties at the three vertices adjacent to the intersected mesh face. For hydrophobicity and electrostatic potentials, the aggregation is a simple average, while in the case of one-hot encoded pseudocenter classifications, the logical “OR” operator is used as aggregation. The result of this process is a set of nine spherical feature maps representing both the shape and physicochemical properties of the pocket. Ignoring potential nonconvexity of surfaces, we postulate that this projection will capture enough information to be useful for the proposed tasks. Maps are sampled using a HEALPix sampling with *N*_side_ = 16 (*n* = 12*N*_side_^2^ = 3072 pixels), and a graph is built using *k* = 20 nearest neighbors with a kernel width *t* set to the corresponding optimum as used by Defferrard et al.^63^

### Training Details

#### General Architecture

For all experiments, we use the same base architecture consisting of four graph convolution (GC) layers each followed by batch normalization, a ReLU activation, and a max pooling layer that down-samples the spherical maps by a factor of four. A global average pooling is added along with a fully connected (FC) layer to produce a final embedding. Global average pooling ensures a rotationally invariant output computing the average across pixel level feature maps. The polynomials of the GC layers are all of order *K* = 3, and the number of channels per layer is 32, 64, 128, and 256. The size of the feature map after average pooling and before it is passed to a fully connected layer is 256.

#### Classification Objective

A multiclass classification training objective is defined using the TOUGH-C1 dataset where the objective is to discriminate between nucleotide, heme, and control binding sites. A fivefold cross validation strategy is used to assess model generalizability, using the same splits defined by Pu et al.,^31^ with performance metrics averaged over the folds. Since the task requires the network to make a prediction based on three ligand types, the FC layer is set to an output size of three and a Softmax activation layer is added to the network. Softmax is defined as follows:
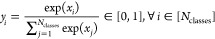
where *N*_classes_ is the number of classes to discriminate, outputting a discretized conditional distribution for the class based on the input and model parameters. The Adam algorithm^67^ is used to optimize the cross-entropy loss defined as follows:
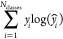
where *N*_classes_ is the number of classes to discriminate, *y* is the ground truth labels, and *ŷ* is the predicted probability that an observation is of class *i.* The learning rate, weight decay, and β1 and β2 hyperparameters are set to 0.05, 0.0, 0.9, and 0.999, respectively. To aid with convergence, a stepped learning rate decay scheme is used where the learning rate is decayed by λ = 0.1 every 25 epochs. We find empirically that batch sizes greater than 32 yield no performance benefit and that the model converges in approximately 60 epochs, taking approximately 15 min per fold.

During training, random rotations are applied to the inputs to enforce rotation invariance and increase the amount of data available to the model. To analyze the importance of certain features, multiple models are trained with different feature combinations. All experiments are evaluated using the receiver operating characteristic (ROC) and the corresponding area under the curve (AUC). The model is further evaluated using a set of steroid-binding sites provided in the TOUGH-C1 dataset. This dataset tests the model’s ability to make predictions on unseen data.

#### Representation Learning Objective

One of the objectives of using machine learning to estimate similarity metrics is to learn a generalizable function that maps a set of input features to a latent representation while also preserving the semantic distance in the input space. This form of learning is particularly useful where the number of target classes is either very large, the number of data points is small, and/or only a small subset of classes is known while training. This learning paradigm aligns well with the binding-site similarity objective, where labeling is an expensive task and the number of classes is not known during training. For example, multiple ligands may bind to the same binding site and many of these will not be known for the purpose of labeling.

A classification objective may not be the best approach for learning binding site representations since the objective enforces the formation of class boundaries in latent space where unknown classes cannot be discriminated. A pairwise relationship between binding sites can be constructed on the basis of shared ligand binding; a representation learning objective in theory will produce representations which can be extended beyond inputs that have been seen during training. Since training models of this nature require large amounts of data and the majority of binding site similarity data are small, handcrafted datasets, we follow Simonovsky and Meyers^29^ using the TOUGH-M1 dataset which represents the largest and most balanced dataset to date.

The model’s objective is to output embedding vectors, rather than discretized probability distributions, where the vector space of structurally and chemically similar pockets is closer than those of dissimilar sites. Of particular importance in this paradigm is that each input is mapped independently to an embedding vector, and the subsequent similarity computation occurs in vector space. In this way, embeddings of graphs can be precomputed and indexed allowing fast nearest-neighbor retrieval. Since an embedding vector output is expected, the FC layer is set to output a vector with a length of 256 and the Softmax activation is discarded.

Multiple loss functions have been proposed for the metric learning task in the computer vision literature utilizing pairs, triplets, or *N*-sets of descriptors. Defining triplets or larger sets of inter-relationships is problematic from a binding pocket point of view where ground-truth relationships for most pairs are unknown. Therefore, we only consider pairs of sites while training, minimizing a margin loss in the following equation.
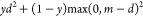
*y* represents the ground-truth relationship and *y* : *y* equals 1 if the two pockets are labeled similar and 0 if not. *d* is the Euclidean distance between the two pocket features, *f*_1_, *f*_2_: *d* = ∥*f*_1_ – *f*_2_∥_2_.

The loss encourages the features of similar pairs to lie close to each other in Euclidean space, while negative pairs are separated by margin *m* > 0. Controlling the value of the margin “loosens” or “tightens” the constraint. We set *m* = 1.0 and minimize using the Adam algorithm with the learning rate, weight decay, and β1 and β2 hyperparameters set to 0.0005, 0.0, 0.9, and 0.999, respectively.

During training, random rotation augmentations are added to both positive and negative pairs to enforce the rotation invariance of the architecture and to increase descriptor robustness. A robust data splitting strategy is essential when working with protein structure to avoid data leakage where a protein structure appears in both training and testing sets. To mitigate this issue, we follow the test/train splitting strategy as implemented in DeeplyTough, where protein structures sharing more than 30% sequence identity are allocated to the same sequence cluster and then allocated to either a training or testing set, according to a random seed. This strategy is used in tandem with a fivefold cross-validation protocol for robust evaluation of generalizability. The maximum number of training pairs per epoch is constrained to a random selection of 25,000 in batches of size 128 to increase efficiency and prevent overfitting. The model converges in 50 epochs taking approximately 2 h per fold.

Using TOUGH-M1 and DeeplyTough, the ROC and the corresponding AUC are reported for a fair and consistent comparison with other similarity algorithms. The final model is further evaluated using the ProSPECCTs dataset where any proteins also occurring in TOUGH-M1 are removed before training according to the aforementioned criteria. To evaluate the model’s utility outside of computing pairwise similarity classifications, the trained model is used to cluster a set of proteins belonging to the kinase family.

## Results and Discussion

### Classification Performance (TOUGH-C1)

During model optimization, different combinations of spherical feature maps representing shape and physicochemical properties were used to identify the most discriminative features (Table 3). Each experiment was performed using a fivefold cross-validation procedure with performance metrics averaged across the folds. For the first three tests, single physicochemical feature maps were considered (charge, hydrophobicity, and pseudocenter features). The performance was acceptable, with pseudocenter features outperforming charge and hydrophobicity by a small margin.

**Table 3:** Test Results of the Classification Task (TOUGH-C1) Using Different Combinations of Feature Maps (Shape and Physicochemical)

input features	mean ACC (combined)	mean AUC (heme)	mean AUC (nucleotide)
charges	0.74	0.89	0.87
hydrophobicity	0.72	0.93	0.88
pseudocenters	0.78	0.94	0.89
shapes, charges	0.78	0.96	0.90
shapes, hydrophobicity	0.75	0.95	0.89
shapes, pseudocenters	0.79	0.94	0.89
shapes, charges, hydrophobicity, pseudocenters	0.81	0.97	0.93

The next three tests incorporated shape information (distance and angles) into the considered feature maps. Incorporation of shape information increased performance when paired with charge and hydrophobicity feature maps yet, surprisingly, when combined with pseudocenters, did not affect performance. The nucleotide class contains flexible molecules with diverse conformations; this may explain why, in this context, shape information adds little to performance compared with physicochemical features. The greater performance for heme-binding sites may also be explained based on conformational flexibility; heme is considerably more rigid than nucleotides, and thus, heme-binding sites also tend to be more structurally similar than nucleotide-binding sites.

**Figure 3:**
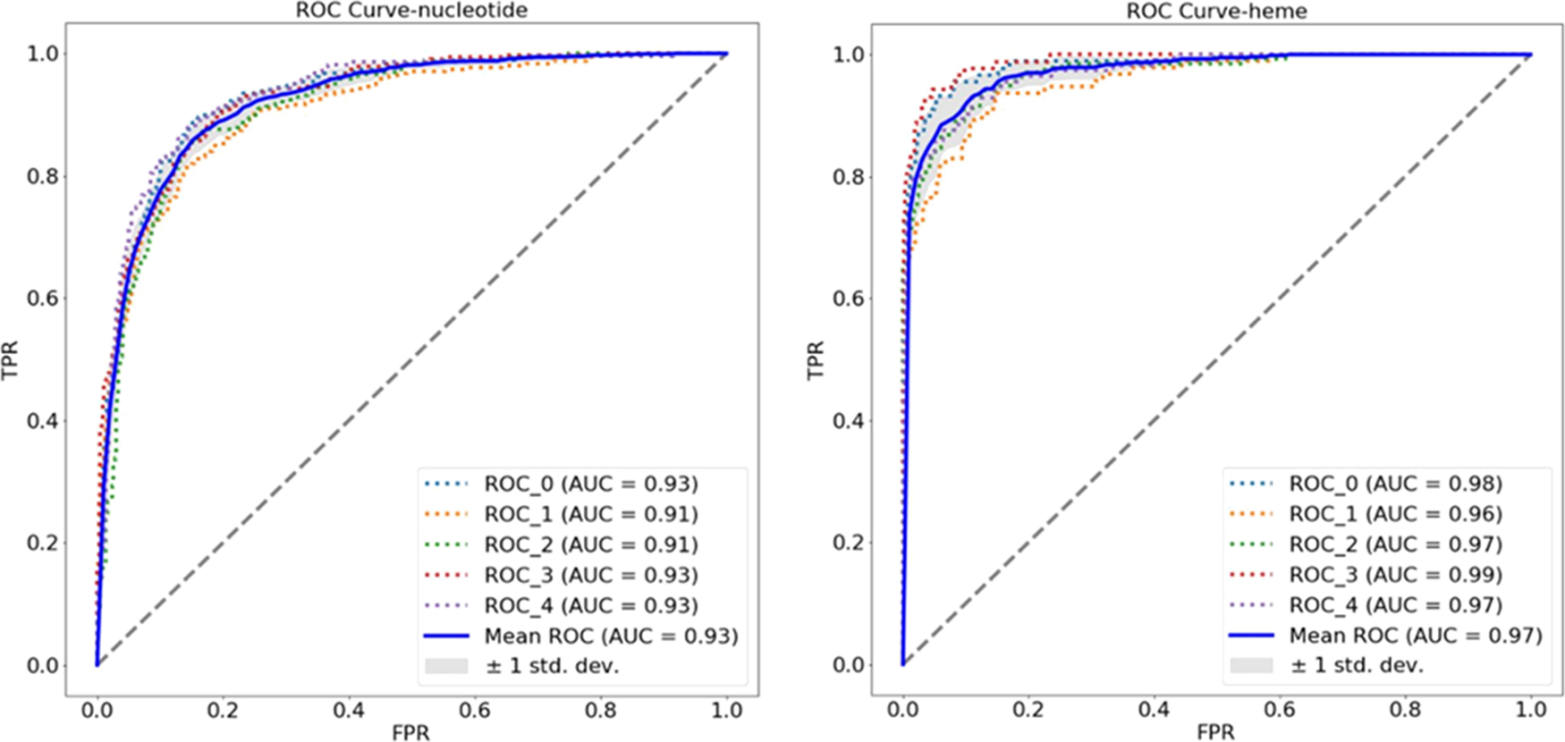
ROC curves for a fivefold cross-validation evaluation on the TOUGH-C1 classification task (nucleotide vs heme vs control).

For the final experiment, all feature maps were considered, displaying the best performance out of all of the combinations, although only by a small margin. For the rest of the experiment, models trained using all feature maps were considered. ROC curves for the fivefold cross validation using all feature maps are shown in Figure 3.

The performance of our models when classifying heme-binding sites is comparable to those reported by DeepDrug3D; AUC 0.974, vs 0.987. However, for the classification of nucleotide-binding sites, our obtained AUC is 0.93, which is less effective than that of DeepDrug3D (0.986). We attribute the performance loss to the spherical parametrization being more sensitive to binding site definition and conformational flexibility. One issue may be due to the ray-casting approach only being able to transform star-like shapes without a loss of information (information is only recorded from the first ray intersection with the molecular surface). This could potentially be remedied using a different spherical projection approach such as conformal mapping.^68^ With this approach, different shape features such as the heat kernel signature^69^ could be used, resulting in less sensitivity to flexibility. DeepDrug3D was further evaluated on a set of steroid-binding sites as a negative control set. BindSiteS-CNN achieves an accuracy of 0.86 on this particular set. We believe that an additional in-batch triplet loss may aid with separating classes further.

Despite a slightly worse performance compared to DeepDrug3D, the results demonstrate that BindSiteS-CNN can learn task-specific binding site representations from feature attributed spherical projection of molecular surfaces. The model and representation are more computationally efficient than 3D voxel-based algorithms, requiring much less memory and training time (1 h 15 vs 3 h for a fivefold cross validation). The learnt representation is also invariant to rotations, a highly desirable property, especially in the case of binding sites where no canonical orientation exists. 3D-CNNs do not share this property; hence, DeepDrug3D requires standardization of the orientation of input structures through alignment of the longest, middle, and shortest principal axes to the *x*, *y*, and *z* Cartesian axes, respectively. This alignment is calculated through the calculation of eigenvectors from the atom positions’ covariance matrix, which does not fully describe the geometric properties of the binding site, occasionally leading to different principal axes for similar shapes. It is expected that when using such approximations for evaluating the similarity between binding sites, an inherent lack of rotation invariance will be a key issue.

### Representation Learning Performance (TOUGH-M1)

Despite promising results on the classification task, the objective is limited to distinguishing between two classes of ligand and a control class. Such a simplistic task is not particularly useful in practice. Extension to multiple ligand classes poses a problem since there is no even distribution of ligand classes in the PDB and a class-based distinction is not simple to construct since structural similarities between ligands do not lend themselves to discrete class separations. We thus train BindSiteS-CNN with a metric learning objective which learns representations which reflect input similarities in metric space. This paradigm of learning minimizes distances between similar sites in metric space while maximizing the distance between dissimilar sites, thus eliminating the issue of requiring class distinctions as the form of hard supervision.

Using the combination of all spherical feature maps, BindSiteS-CNN was trained using the TOUGH-M1 dataset as described where the pocket is determined through the pocket prediction method FPocket.^9^ BindSiteS-CNN achieves a mean AUC of 0.86 ± 0.003 compared to DeeplyTough 0.91 ± 0.003. Interestingly, when trained using the pocket defined as atoms within 6 Å of a bound ligand, the performance increases to AUC 0.89 ± 0.002, highlighting an increased sensitivity to the input pocket definition compared with volumetric methods. Compared with the next best performing methods, both deep learning-based methods significantly outperform other binding site similarity tools: SiteEngine (AUC 0.732), G-LoSA (0.694), PocketMatch (0.644), and APoc (0.644). As noted by Simonovsky and Meyers,^29^ analysis of BindSiteS-CNN results indicate that false negatives and false positives may indicate questionable ground-truth labels in the dataset, where shared binding of molecules may be attributed to the promiscuity of the molecule rather than an indication of pocket similarity. Furthermore, construction of negative pairs of binding sites is a particularly difficult task since the lack of a structural/experimental conformation of binding does not mean that binding related ligands is unfeasible. Ideally, negative pairs should be informed by activity measurements. However, a lack of such annotations (on a large scale) precludes this type of construction, especially in the case of machine-learning-based approaches.

### ProSPECCTs Datasets

Machine-learning-based procedures are prone to reflecting biases present in training data, especially in the case of protein structural data where splitting strategies are not straightforward due to a nondiscrete structural landscape. We expect BindSiteS-CNN to be less susceptible to data leakage in this manner since only the surface of the pocket is considered, as opposed to a voxel grid over the entire pocket which consequently contains parts of the protein structure not involved in the binding of the ligand and hence may be considered not relevant to the protein–ligand interaction. It is difficult to say what influence the nonrelevant information may have on the final predictions.

**Figure 4:**
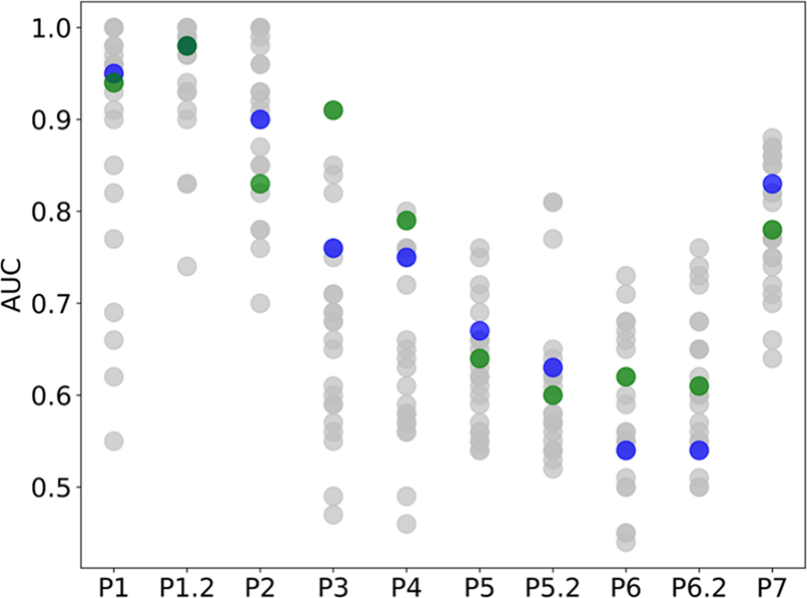
AUC value comparison for BindSiteS-CNN. The AUC of BindSiteS-CNN is in green, DeeplyTough is in blue (the same value as BindSiteS-CNN for P1.2), and the AUCs of other binding site similarity tools are in gray. All were calculated on subsets P1 to P7 of the ProSPECCTs dataset benchmark collection.

Due to these potential biases, methods must be evaluated on independently constructed datasets which may also vary in the labeling procedure and binding site definition. We evaluate the trained BindSiteS-CNN model (predicted pocket parameterization) with the ProSPECCTs dataset. It consists of 10 separate datasets, and each designed to test a different aspect of a binding site similarity tool. Since ligand information is available in all datasets, pockets are defined by all atoms within 6 Å of the heavy atoms of a bound ligand. AUC scores are shown for each ProSPECCTs dataset in Table 4 and additionally visualized in Figure 4, which compares the rank of our method against the ranks obtained by the 23 tools described in the DeeplyTough paper.

**Table 4:** AUC Values for BindSiteS-CNN on Each of the 10 ProSPECCTs Datasets and Its Ranking Compared to 23 Other Binding Site Similarity Evaluation Tools

	P1	P1.2	P2	P3	P4	P5	P5.2	P6	P6.2	P7
reference range^a^	0.55–1.00	0.74–1.00	0.70–1.00	0.47–0.85	0.46–0.80	0.54–0.76	0.52–0.81	0.44–0.73	0.50–0.76	0.64–0.88
BindSiteS-CNN	0.94	0.98	0.83	0.91	0.79	0.64	0.66	0.62	0.61	0.78
rank (in 24 tools)	11	11	20	1	2	11	4	6	10	11

a The reference ranges and ranks are constructed from information accessed from ProSPECCTs and DeeplyTough. The rank is inclusive of BindSiteS-CNN.

Datasets P1 and P1.2 are designed to assess the sensitivity of tools to the definition of a binding site. Dataset P1 assesses this through the comparison of binding sites extracted from proteins with identical sequences yet binding chemically distinct ligands. BindSiteS-CNN achieves an AUC of 0.94, demonstrating a reasonable robustness to input definition. Dataset P1.2 restricts comparison to identical ligands. Promisingly, the AUC increases to 0.98, highlighting that the model is robust when considering identical proteins.

Dataset P2 uses ensembles of nuclear magnetic resonance structures to assess sensitivity to binding site flexibility. While an AUC of 0.83 is not a bad result per-se, BindSiteS-CNN ranks as one of the lowest of the evaluated tools. This observation highlights an inherent sensitivity to conformational variability in a protein structure. Such a sensitivity may be desirable in certain applications. Sensitivity is further highlighted through the evaluation of datasets P3 and P4. These evaluate a tool’s ability to discriminate between sites which differ by five artificial mutations, with P3 considering mutations leading to a change in physicochemical properties and P4 considering mutations leading to both a change in physicochemical properties and in shape. BindSiteS-CNN displays excellent performance here, with AUCs of 0.91 and 0.79, respectively, also ranking 1st and 2nd out of all tools for these tasks.

P5 and P5.2 represent datasets for shape similarity analysis between the ligand and binding site. The dataset contains 10 different ligand classes, with the P5.2 version also including phosphate-binding sites. Performance on both sets ranks within the top 50% of tools (AUC 0.64–0.66), with the performance on P5.2 being ranked 4th. We attribute the better performance to the inclusion of phosphate in the negative-image surface parameterization, and a single phosphate (PO_4_) should be easy to distinguish in this regard being such a small, almost spherical molecule. False positives on this particular set occur between similar nucleotides such as AMP and ATP, which often share similar shape and similar binding-site features. Indeed, tools that perform well on these datasets consider protein–ligand interactions or size explicitly and hence find it easier to distinguish such examples. The inclusion of a size-based scoring function increases performance significantly on this set, suggesting that sites may be differentiated by size alone rather than physicochemical features.^70^

P6 and P6.2^71^ comprise pairs of dissimilar proteins, with similar local environments, binding to identical ligands. P6.2 excludes cofactors. BindSiteS-CNN again ranks in the top 50% (AUC 0.62–0.61), although results on this set should be considered with a pinch of salt due to the small size of the dataset and unconvincing results from the majority of evaluated tools. The final dataset P7 measures the recovery of known binding site similarities compiled from literature sources. With an AUC of 0.78, our method achieved a moderate performance ranked in the top 50% of all tools evaluated.

In summary, BindSiteS-CNN displays good performance across the ProSPECCTs datasets, and its observed sensitivity to minor changes in physicochemical properties on the molecular surface highlights its potential usage applications. We propose that, with a consistent binding site definition, BindSiteS-CNN will be a good method for distinguishing protein-binding sites within a particular protein family based on small variations. The ability to distinguish between minor variations may have applications in inferring selectivity patterns in binding. It is also interesting to note that BindSiteS-CNN outperforms or matches the performance of DeeplyTough, the only other deep-learning-based tool, in six out of ten of the ProSPECCTs datasets, despite being more efficient and not requiring extra loss functions to maintain stability while training. It would be interesting to see whether a meta-classifier using the outputs of multiple machine-learning-based methods, with different input parameterizations, would improve retrieval performance.

### Classification of ATP-Binding Sites of Protein Kinases

The protein kinases are among the most studied druggable targets, especially in searching for anticancer therapies.^72^ Their active site, the ATP-binding site, exhibits remarkable structural variation as observed in the large number of PDB structures, even though all the kinases have the same substrate ATP. The medicinal effort for the past 20 years has seen a high diversity of synthetic ATP mimetics/inhibitors for different kinases.

A drug’s specificity and selectivity are very important when targeting in the drug discovery process.^49^ Especially challenging is the active form of the pocket. Many successful efforts have been published trying to classify the structures and their interactions with inhibitors.^48,49^

ATP is the natural substrate of the kinases. It binds in the deep catalytic cleft formed between the N- and C-lobes, with its adenine ring forming hydrogen bonds with the kinase. Kinase activity is regulated by a conserved activation loop, formed by the DFG and APE motifs, which is highly flexible. The term “DGF-in” refers to a usually active conformation, whereas “DFG-out” is an inactive one. The structures in the PDB reflect this flexibility in the wide range of conformations observed, and it is this flexibility that makes automatically classifying the kinases into their subfamilies such a challenge, as well as complicating efforts in drug design.^46−50^

Here, we compared the binding conformations of the protein kinase ATP-binding sites using BindSiteS-CNN to test if our method can classify kinases based on the learned features of their binding sites. Our set of kinases contains either the “DFGin/out” motif (for kinase activation) or the “alphaC in/out” conformation, as stated in the dataset section. There are 1264 structures in total, covering 26 families within 7 groups. This information is available in the Supplementary Information section. These structures are all co-crystallized with different inhibitors in the same binding site, such as ATP, and other chemical structures. The set of kinase structures includes (see Table S1) PDB structures of many different subfamilies as well as different structures of the same family. The set reflects the flexibility of the kinase-binding sites. As the set also contains DFGin/out, the flexibility of the activation loop is also considered.

**Figure 5:**
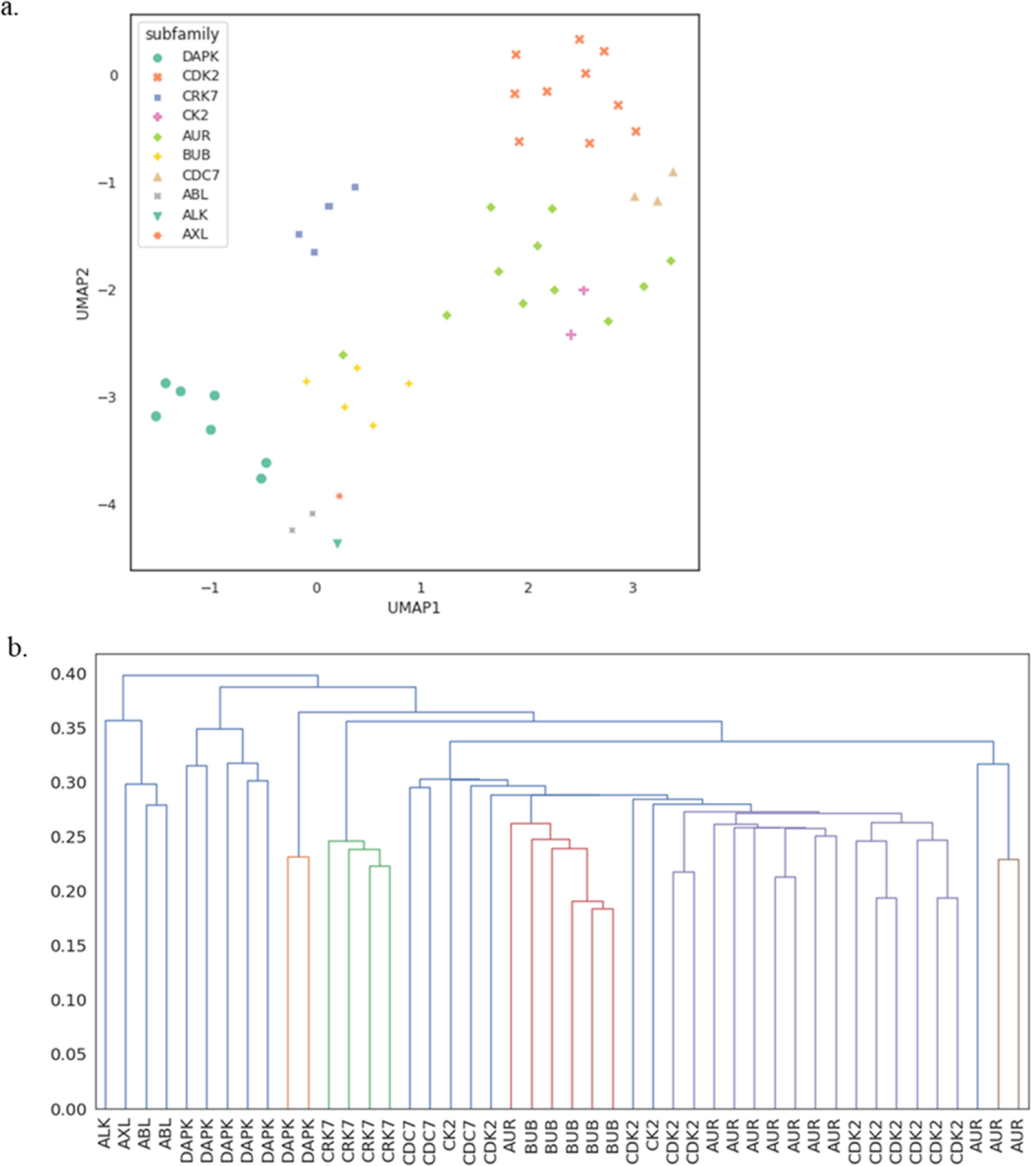
Results for kinases with bound ATP. (a) UMAP visualization of learnt binding site descriptors. Examples labeled by the subfamily. (b) Dendrogram illustration of the hierarchical clustering of descriptors.

We have used UMAP^73^ to visualize the descriptor space learnt by BindSiteS-CNN. First, we selected a subset of the inhibitors that are only ATP. This was to test if our program can distinguish/classify subfamilies based on learnt features even though they have the same inhibitor, ATP. Figure 5a shows the clustering results of 10 subfamilies. The results show that even though they have the same ligand, their binding sites are different. A co-crystallized inhibitor/protein structure does not necessarily reflect that the binding site contains only features influenced by the bound compound. In addition, given that the kinase-binding sites are quite flexible among the subfamilies, as well as within the same subfamily, it was reassuring that our method can still group them into the correct subfamilies. This reflects that, even though the inhibitor is ATP, the flexibility of kinase-binding sites is highly variable. The flexibility comes from different residues lining the binding site as well as the movement of the activation loop (DFG loop).

**Figure 6:**
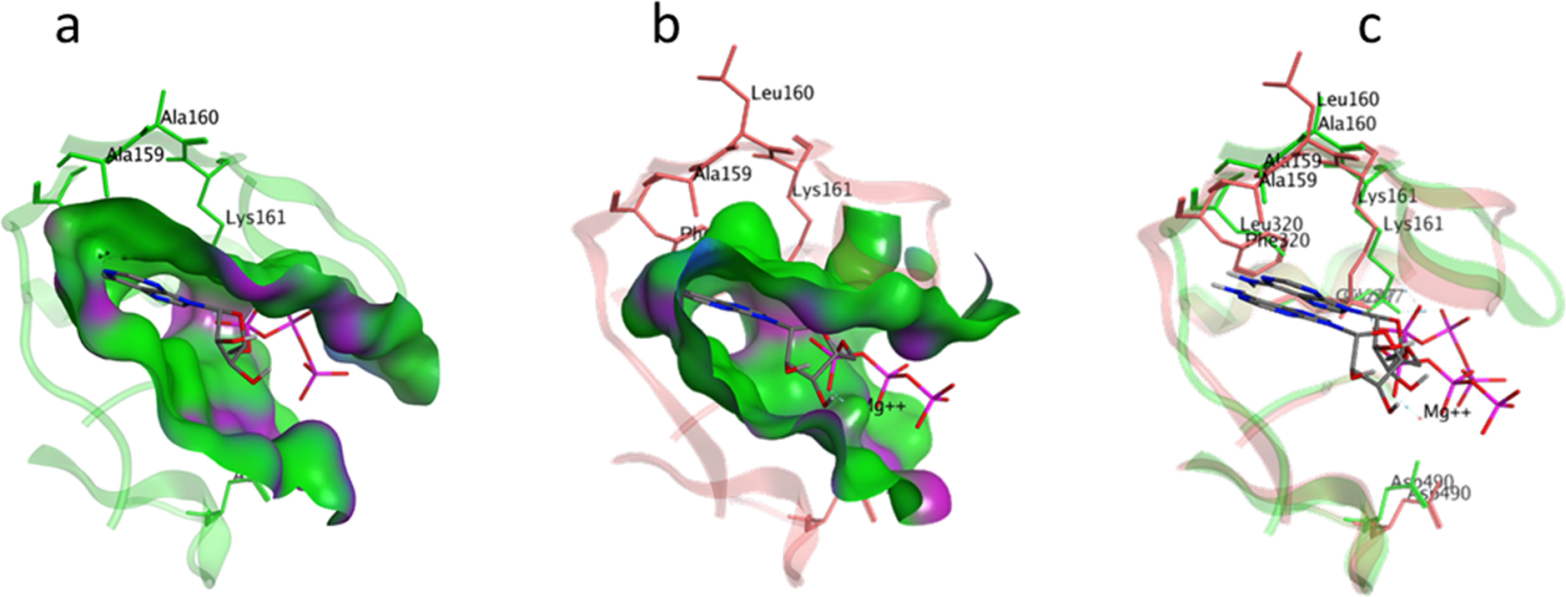
Flexibility of ATP kinase-binding sites. (a) DAP-kinase-related protein DAPK (PDB 2YAA). (b) Cyclin-dependent kinases 2 CDK2 (PDB 4EOJ). The color of the protein surface is mainly based on hydrophobic (green) and polar and hydrogen bonding (purple). (c) Overlap of the two proteins based on structural alignment. Note that the most dissimilar amino acids are Leu320 (DAPK) to Phe320 (CDK2).

In the next experiment, we wanted to evaluate if our method can cluster our set of kinase structures into the family and group. The dendrogram (Figure 5b), resulting from a hierarchical clustering of the learnt descriptors, also reveals that the similarities are consistent with the identity of the kinase subfamilies, with only a small number of mislabeled examples. In Figure 6, the surface comparison is shown graphically for two proteins, DAPK and CDK2. The binding sites of these proteins occur in different clades in the dendrogram in Figure 5. The structures are aligned based on protein structure and not using their inhibitors (ATP). In Figure 6a,b, the surfaces are quite different, despite similar amino acid compositions. This difference can be attributed to a change of Leu320 (DAPK) to Phe320 (CDK2). In Figure 6c, the loops from the two structures cannot be overlayed well, reflecting again the flexibility of the kinase-binding sites in different subfamilies. In general, this method can distinguish these features.

**Figure 7:**
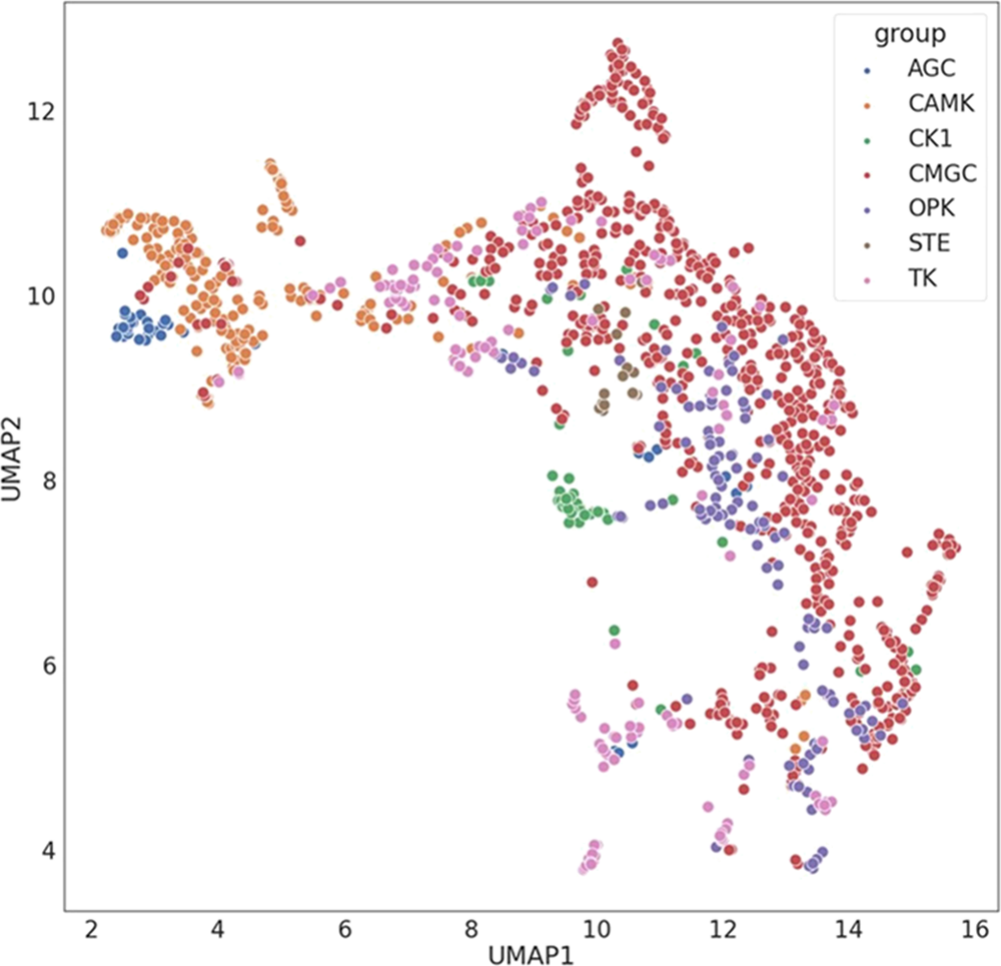
UMAP visualization of learnt binding site descriptors of all selected kinases. Examples are labeled by the definition of the group.

Next, we performed a UMAP dimensionality reduction calculation on all the structures. Figure 7 shows the distribution of the descriptors for the seven different groups of kinases. We can observe that the AGC, CAMK, and CK1 groups form tight clusters, whereas the CMGC and OPK groups are more widely dispersed and more intermixed. The most dispersed distribution is that of the TK group.

**Figure 8:**
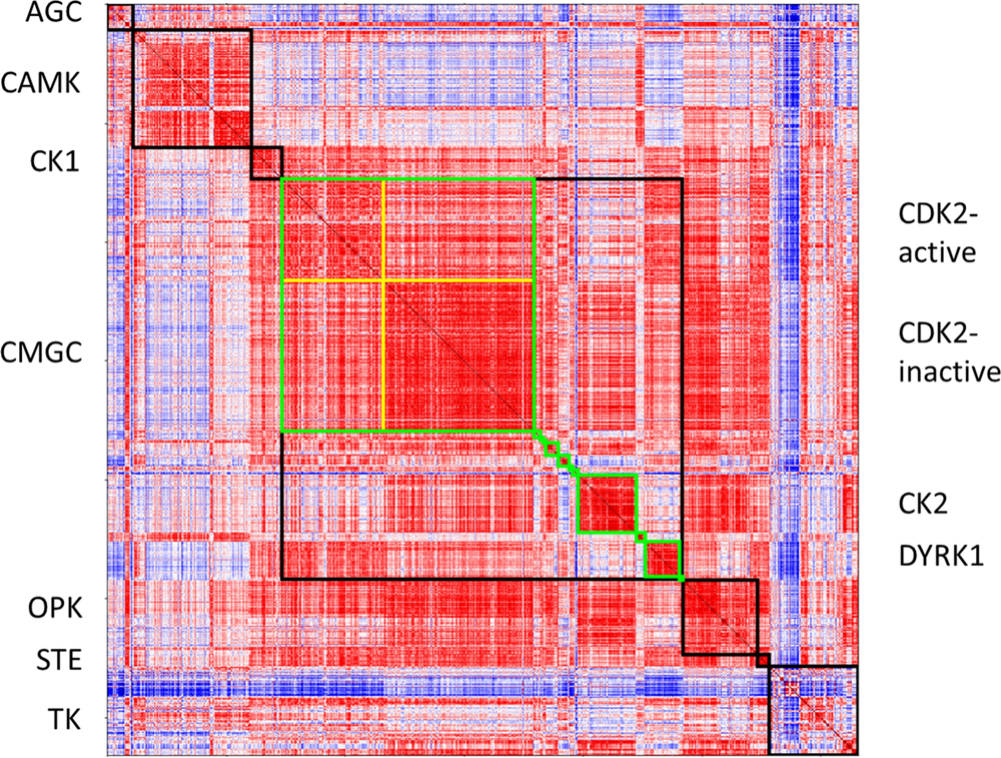
Distance matrix of kinase embeddings. The colors represent the pairwise distances between learnt binding site descriptors: the red regions represent high similarity, whereas the blue regions represent high dissimilarity. The labels on the left identify the various kinase groups, with black squares showing the all-against-all distances within each group. The labels on the right identify the subfamilies of the CMGC group, with the green squares outlining the distances between their members. The yellow borders divide the two main states of CDK2 (active/inactive).

Figure 8 shows the descriptor-based similarity of the structures. Here, the pairwise Euclidean distance between binding sites is used for similarity measurement. In Figure 8, the red regions are more similar (the darker red being the most similar), while the blue regions are more different (the darker blue is the most dissimilar). Figure 8 shows further analysis of the largest group we tested, CMGC, in green squares. The results show that our method can capture the internal similarity of the same subfamily, despite there being sidechain flexibility in the binding site as each protein is represented by several PDBs. A previous comprehensive study of drug binding to different proteins has pointed out that binding between ligands and protein-binding sites has a weak correlation to the conformational flexibility of the binding sites.^13^ Further observation of CDK2 reveals a significant difference in the descriptors of the binding sites of the active and inactive forms. This is consistent with the very good sensitivity to physicochemical properties shown by BindSiteS-CNN and provides the possibility to apply our method to protein functional and characteristic analysis.

## Conclusions

In this study, a spherical CNN applied to spherical projections of binding site surfaces was applied for the classification and similarity analysis of protein-binding sites. Training on the TOUGH-C1 dataset of protein-binding sites demonstrated the ability of the graph-based spherical CNN to learn from binding pocket features. This also reflected how well the obtained surface features describe the protein-binding sites. Parallel experiments based on different combinations of the feature types gave the best combination while verifying the contribution of the features.

Metric learning models were trained using the TOUGH-M1 dataset to learn informative global descriptors of protein-binding sites. The pairwise distances between these descriptors can be used as a basis for scoring the similarity of protein-binding sites. The ability of the obtained models to analyze various aspects of binding site similarity was validated using the independent validation datasets of ProSPECCTs.

BindSiteS-CNN performed well when comparing binding sites with different physicochemical properties. Although our models using spherical CNNs do not outperform all 23 tools on the ProSPECCTs datasets, their ranking is better than most in nearly all. The results, therefore, provide a good proof of concept of the method.

The kinase case study shows that the method has the potential to capture even the difference between different active states of the same kinase subfamily. The trained models could be used to search for local similarity in binding sites of completely unrelated proteins. However, the AUCs for our models on the P6 and P6.2 datasets are fairly low, albeit not the worst. This suggests that our method gives less confident results when applied to unrelated proteins and is more effective within families rather than between families. Nevertheless, this still has great potential for applications in the analysis and prediction of the off-target side effects of drugs, drug repurposing, and protein function prediction. On the other hand, these models may also be used for large-scale inter- and intra-group analysis of protein families. This local characteristic-based observation is expected to help discover new associations between different proteins in terms of physicochemical properties and biological functions in the future.

## Supporting Information Available

Grouping information for the kinase set (PDF)

## Notes

The training datasets (TOUGH-C1, TOUGH-M1, and ProSPECCTs) are publicly available. The kinase set is presented in the Supporting Information section. The method described here was implemented using Python 3.7 and the PyCharm Python Integrated Development Environment. The code and description of procedures are available on GitHub - https://github.com/Jing9558/BindSiteS-CNN
