# Supplementary material for: Classification of Protein-Binding Sites Using a Spherical Convolutional Neural Network
Source: J Chem Inf Model. 2022 Nov 7;62(22):5383–96. doi: 10.1021/acs.jcim.2c00832 (PMC9709917; doi:10.1021/acs.jcim.2c00832)
Supplement: Supplementary file 1 — ci2c00832_si_001.pdf [file ci2c00832_si_001.pdf]

Supporting Information

Classification of Protein Binding Sites using a Spherical Convolutional Neural Network

Oliver B. Scott, Jing Gu, and A.W. Edith Chan

Division of Medicine, University College London, Gower Street, London WC1E 6BT, UK

Email: edith.chan@ucl.ac.uk

Table S1. Grouping Information for kinases

Group	Sample size	Family	Sample size	Subfamily	Sample size
AGC	43	AKT	30	AKT	30
		GRK	8	BARK	8
		PKC	5	ALPHA	5
CAMK	199	CAMK1	3	CAMK1	3
		CAMK2	9	CAMK2	9
		CAMKL	117	AMPK	11
				CHK1	106
		CASK	8	CASK	8
		DAPK	60	DAPK	58
				DRAK	2
		DCAMKL	2	DCAMKL	2
CK1	52	CK1	52	CK1-D	38
				CK1-G	14
CMGC	675	CDK	490	CDK2	424
				CDK4	11
				CDK5	5
				CDK7	4
				CDK8	21
				CDK9	18
				CRK7	7
		CDKL	8	CDKL	8
		CK2	98	CK2	98
		CLK	15	CLK	15
		DYRK	64	DYRK1	58
				DYRK2	6
OPK	125	AUR	114	AUR	114
		BUB	5	BUB	5
		CDC7	6	CDC7	6
STE	20	STE11	20	ASK	20
TK	150	ABL	57	ABL	57
		ACK	10	ACK	10
		ALK	30	ALK	30
		AXL	10	AXL	10
		CSK	2	CSK	2
		DDR	11	DDR	11
		EGFR	30	EGFR	30

AGC - Protein Kinase A, G, and C families (PKA, PKG, PKC)

AKT – Protein kinase B (PKB)

GRK – G protein-coupled receptor kinases

BARK – Beta-adrenergic receptor kinase

PKC – Protein Kinase C

ALPHA – Protein Kinase C alpha

CAMK – Calcium and Calmodulin-regulated kinases

CAMKL – CAMK-like

AMPK – AMP-activated protein kinase

CHK1 – Checkpoint kinase 1

CASK – calcium and calmodulin-dependent serine protein kinase

DAPK – Death-associated protein kinase

DRAK – DAP kinase-related apoptosis-inducing protein kinase

DCAMKL – Doublecortin and CaM kinase-like kinase

CK1 – Casein kinase 1

CMGC – cyclin-dependent kinases (CDKs), mitogen-activated protein kinases (MAP kinases),glycogen synthase kinases (GSK) and CDK-like kinases

CDK – Cyclin-dependent kinases

CRK – Adapter molecule crk

CDKL – CDK-like kinases

CK2 – Casein kinase 2

CLK – CDC2-like kinases

DYRK – dual-specificity tyrosine-regulated kinases

OPK – Other protein kinases

AUR – Aurora kinases

BUB – Mitotic checkpoint serine/threonine-protein kinase BUB (budding uninhibited by benzimidazoles)

CDC7 – Cell Division Cycle 7 – related protein kinase

STE – Serine/threonine kinases

ASK – Apoptosis signal-regulating kinase

TK – Tyrosine Kinase

ABL – Tyrosine-protein kinase ABL1

ACK – Activated Cdc42 kinase

ALK – Anaplastic lymphoma kinase

AXL – Tyrosine-protein kinase receptor UFO

CSK – C-terminal Src Kinase

DDR – Discoidin domain receptor

EGFR – Epidermal growth factor receptor
